# Structured Counselling and Regular Telephonic follow up to improve Referral flow and compliance in Nepal for Diabetic Retinopathy(SCREEN-D Study): a randomised controlled trial

**DOI:** 10.1186/s12913-024-10647-3

**Published:** 2024-02-10

**Authors:** Binita Bhattarai, Hari Bahadur Thapa, Sandip Bashyal, Sarasawati Khadka Thapa, Sirshendu Chaudhuri, Varun Agiwal, Hira Pant, Samiksha Singh, Hemant Mahajan

**Affiliations:** 1 Lumbini Eye Institute and Research center, Siddharthanagar, Lumbini Nepal; 2 Indian Institute of Public Health, Hyderabad (IIPH-H), Hyderabad, India; 3 Lumbini Eye Institute and Research Centre, Bhairahawa, Nepal

**Keywords:** Compliance, Counseling, Diabetic retinopathy, Nepal, Referral systems

## Abstract

**Background:**

Diabetic Retinopathy (DR) is an emerging public health issue, leading to severe visual impairment or blindness. Early identification and prompt treatment play a key role in achieving good visual outcomes. The objective of the study was to estimate the effectiveness of SCREEN package on improving referral compliance from peripheral centres to a tertiary eye centre in Nepal.

**Methods:**

In this facility-based cluster-randomized trial, ten out of 19 referring centres of the tertiary eye care centre in Lumbini zone, Nepal were randomized into intervention and control groups. A SCREEN packagewereprovided as intervention for DR patients who require advanced treatment in the tertiary centres and was compared with the current practice of the control arm, where structured counselling and follow-up mechanism are absent. Compliance was estimated by a weekly follow-up between the referring centre and the referred hospital.

**Results:**

We recruited 302 participantsof whom 153 were in the intervention arm. The mean age of the participants was 57.8 years (Standard deviation [SD]±11.7 years). With implementation of SCREEN package71.2% (*n*=109) in the intervention group and 42.9% (*n*=64) in the control group were compliant till three months of follow-up (Difference 28.3%, 95% CI: 17.6- 39.0, *p*<0.05). Compliance was 43% (*n*=66) with counselling alone, and 66% (*n*=103) with first telephonic follow-up in the intervention arm. The mean duration to reach the referral centre was 14.7 days (SD± 9.4 days) and 18.2 days (SD± 9.1 days) in the intervention and the control arm, respectively (Difference 3.5 days, 95% CI: 0.7 to 6.4 days).

**Conclusions:**

Counselling& follow-up to patients is the key factor to improve the utilization of the health services by patients with DR. Health systems must be strengthened by optimizing the existing referral structure in Nepal.

**Trial registration:**

ClinicalTrials.gov Protocol Registration and Results System, ClinicalTrials.gov Identifier: NCT04834648, 08/04/2021.

**Supplementary Information:**

The online version contains supplementary material available at 10.1186/s12913-024-10647-3.

## Background

Changes in lifestyle and increased life expectancy have escalated the burden and effects of diabetes worldwide [1]. Epidemiological studies have shown that approximately one out of three patients suffering from Diabetes Mellitus (DM) develop Diabetic Retinopathy (DR) over time. One in 10 diagnosed with DR has Proliferative Diabetic Retinopathy (PDR) and Diabetic Macular Edema (DME). Globally, DR has remained a leading cause of avoidable blindness among the populations over 50 years of age [2].Pooled estimate suggests that globally, the prevalence of DR among diabetic populations is 22.27% and the estimated number of adults with DR is one billion [3, 4]. It is expected that the absolute number may cross 1.6 billion by the end of 2045. DR ranks as the fifth most common cause of global blindness and global moderate and severe visual impairment (MSVI) [5].

DR is broadly classified based on classical retinal lesions: Non-Proliferative Diabetic Retinopathy (NPDR), PDR and DME. NPDR is classified into mild, moderate, and severe NPDR [3]. Though asymptomatic initially, irreversible visual impairment is seen in the proliferative and macular involvement stages. Therefore, early detection of PDR is crucial at the pre-proliferative stage by regular eye examination to prevent vision loss [6].Timely treatment of DR can reduce the risk of vision loss by 60% [3, 5–10].

Several studies showed low awareness about the damaging effects of DR on visual acuity amongst people with diabetes [3, 7].Educating and counseling patients is the cornerstone for improving compliance. Simultaneously, referral protocols with strong communication and support systems are essential to ensure timely diagnosis and management among the screened population. A study from Tanzania suggests that improving the clarity of the referral process by explaining the reasons for referral, treatment costs, and possible health benefits to the DR patient substantially improved the visits to the higher centresfor availing retinal services [11].

Various studies from Nepal suggest that the prevalence of DR ranges between19% and 78% [12]. In a cross-sectional study done in two cities of Nepal the prevalence of non-proliferative DR, proliferative DR and complete vision loss are 9.1%, 0.5%, and 0.3%, respectively [12]. Among patients with diabetes at Lumbini Eye Institute and Research Centre (LEIRC), a study showed that the hospital magnitude of NPDR, PDR and advanced diabetic eye disease (ADED) was 69%,31% and 3%, respectively. This indicates poor compliance with referral advice for patients referred from the primary level to the tertiary level [13].

LEIRC is a tertiary eye care centre in the western region of Nepal. Despite having 19 peripheral referral centrescovered under the LEIRC umbrella, there is a poor inflow of patients with DR to the tertiary centre. There is a lack of systematic referral, communication and support system to ensure compliance, continuity of timely care and appropriate management.

We, the same research team, conducted a problem tree analysis (Unpublished) and found the main problem to be the long waiting time and lack of a proper referral system. We mapped the referral flow of the DR patients and identified the barriers at the level of the participants, the referring centre and the referral centre. (Supplementary figures 1 and 2) We identified that long waiting time at the referred centre, lack of knowledge about DR, its impact on vision and different treatment options are the major barriers. We also found administrative challenges in the referred hospital like absence of referral registers, lack of fast-track system for the referred patients and mis-utilization of resources by repeated examinations that were already done in the referring hospital. We identified appropriate solutions through comprehensive literature review and brainstorming to improve the referral compliance. These solutions were systemically arranged in the form of an intervention package (SCREEN package), which were tested through this cluster-based randomized controlled trial.Hence, we planned an intervention package that included structured counseling, telephonic follow up, and communication across referring and referral facilities to improve the DR outcome.

The primary objective of our operational research study was to estimate the effectiveness of intervention on improving referral compliance from peripheral centres to a tertiary centre in Nepal. We also estimated the difference in time (in days) taken by the compliant patients to reach the tertiary centre.

## Methods

### Study design

This is an operational research study using cluster randomized controlled trial.

### Study participants

All the DR patients diagnosed in the peripheral eye centres were eligible to participate in the study.

#### Inclusion criteria

All DR patients who are referred from one of the 19 peripheral eye centre to LEIRC and who are willing to participate in the study.

#### Exclusion criteria

DR patients who have received multiple treatments from other centres, and DR patients who directly visit the referral centre (direct walk-ins).

### Study setting

**Fig. 1:**
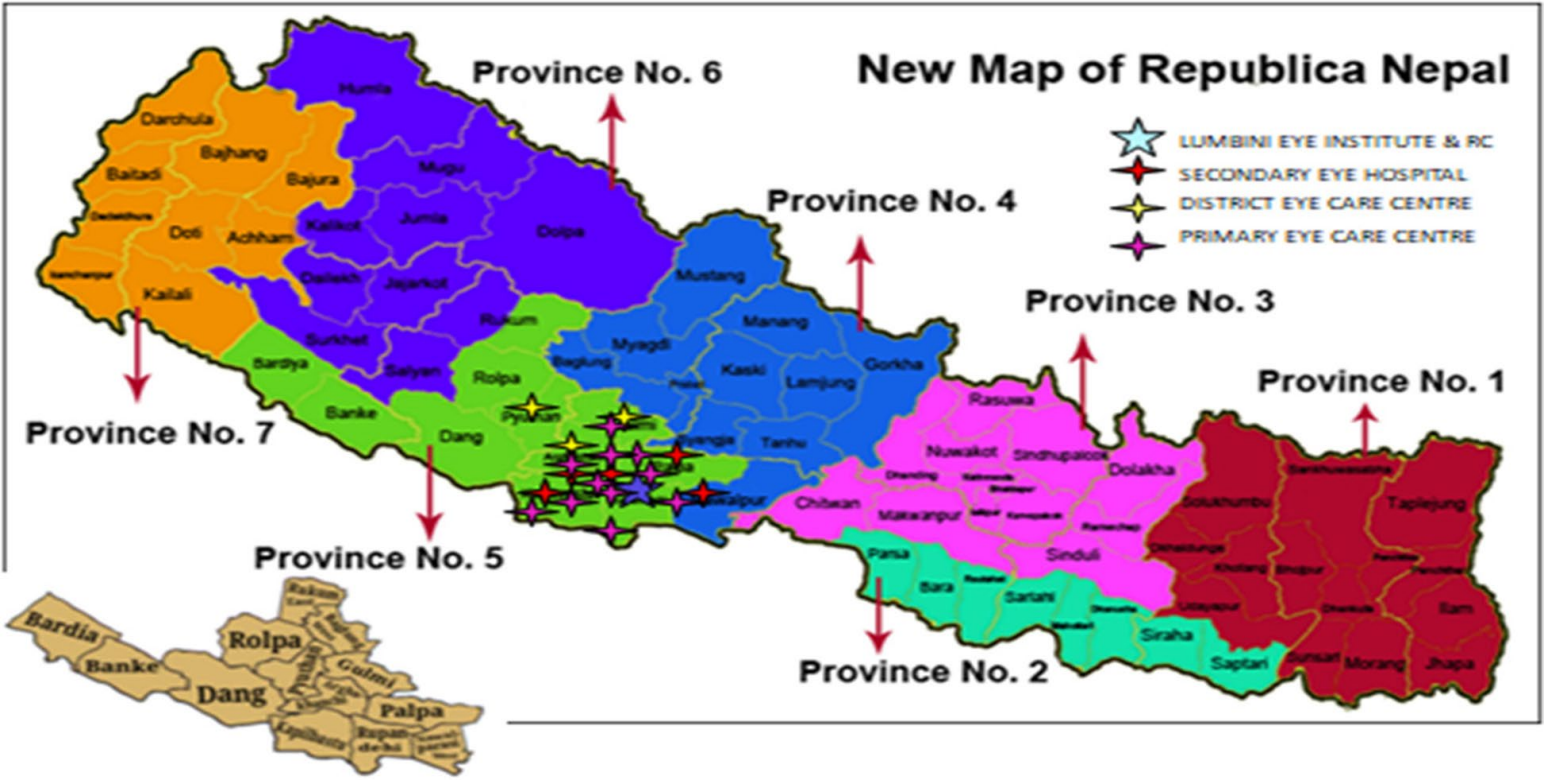
Map of LEI and its peripheral centres

We conducted this study based in LEIRC (referral centre) and its nineteen referring centres located in the Western part of Nepal. The referring centres include five secondary eye care centres (SECC), three district eye care centres (DECC) and eleven primary eye care centres (PECC) (Fig. 1).

The referral centre has several sub-specialty services. It is the only retinal centre in this region of Nepal. The Retina department is well equipped and served by three retina specialists and a counsellor. A secondary eye hospital has an ophthalmologist and three ophthalmic paramedical personnel (optometrist/ophthalmic assistant). PECC and DECC have ophthalmic paramedical personnel (optometrist/ophthalmic assistant). In these settings, the general ophthalmologist and the ophthalmic paramedical personnel are trained to diagnose DR using instruments like slit lamps and direct ophthalmoscope.

### Study duration

One year between May 2021and May2022

### Interventions

**Fig. 2:**
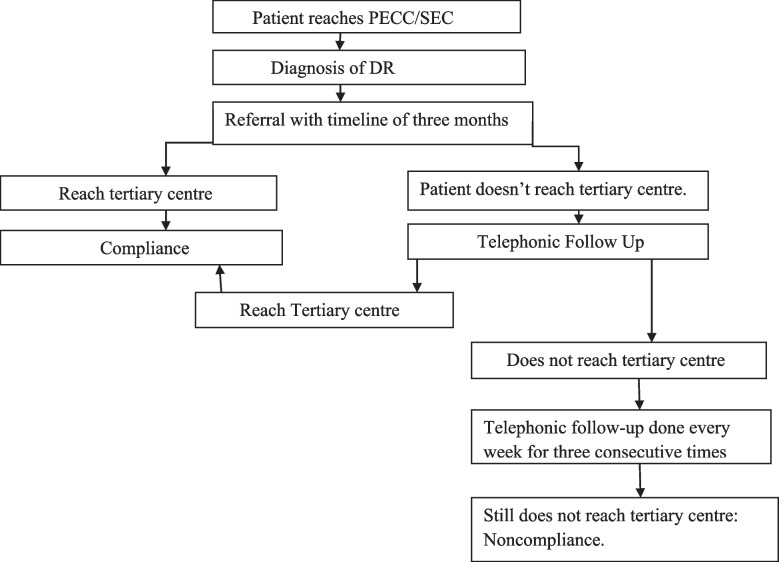
Participants timeline

The intervention package included structured counselling and telephonic follow-up, referral communication, and fast-tracking of referred cases of DR. We trained two members (head of the centre and one ophthalmic staff) from each referring centre for counselling the DR patients. We provided a checklist to the counsellors to maintain the uniformity in training. The checklist included verbal counselling of the participants about the impact of DR on vision, nature of vision loss, treatment options, and cost; guidance on transport to referral centre from their place, contact number of the designated person at referral centre, provision of a health education material; and maintenance of a dedicated referral register for DR patients. As a part of the intervention, the counsellors followed up monthly with all the patients to check the compliance. If non-compliant, the counsellors documented the reason(s) for non-compliance and reiterated the importance of timely treatment (Fig. 2). The counsellors called-up the non-compliant participants for a maximum of three times at different time intervals. For participants lost to follow-up, the counsellors tried to understand and record the reasons. The counsellors also followed up with patients who were clinically reviewed at the referral centre and needed support at the community level, as per the feedback from the referral centre.

The intervention was compared with the current practice. As per the current practice, DR is provisionally diagnosed at the peripheral centre. The counsellors recorded the participants’ information, briefed them about the changes in the retina and referred them to LEIRC for definitive diagnosis and management. They also provided health education material that had diagrammatic explanations of the changes in the retina due to DR and its impact on vision. There was no provision for other components like follow up as described in the intervention arm.

### Referral communication and feedback between the peripheral centre and Referral hospital (For both arms)

We developed a fast-track system at the referral centre. The out-patient department (OPD) ticket at the referral centre got stamped as ‘REFERRED’ at registration. The registration section directed the participants to visit the General Ophthalmologist and skipped the routine eye examination by an optometrist at OPD, thus saving time to reach the ophthalmologist. We used the existing IT-based communication system to record all referred cases at the referring centre. A referral manager updated the counsellors on the referred participants they received, their diagnosis and treatment detail. All the participants underwent treatment as per the hospital protocol.

### Outcome variables

#### Primary outcome

Proportion of referral compliance in the two arms. We defined a participant as referral compliant if the patient reached the LEIRC within three months of referral.

#### Secondary outcomes

Time taken in days to reach the LEIRCin both the groups.

### Sample size & participant recruitment

Previous evidence showed that the proportion of patients who comply with advice for referral is 25%.(11) To detect an absolute improvement of 25%referral compliance in the intervention arm, we estimated a sample size of 148 in each group (allocation ratio 1:1) in both the arms with 80% power at a 5% significance level, assuming a design effect of 2, and the non-response rate of 20%.

**Fig. 3:**
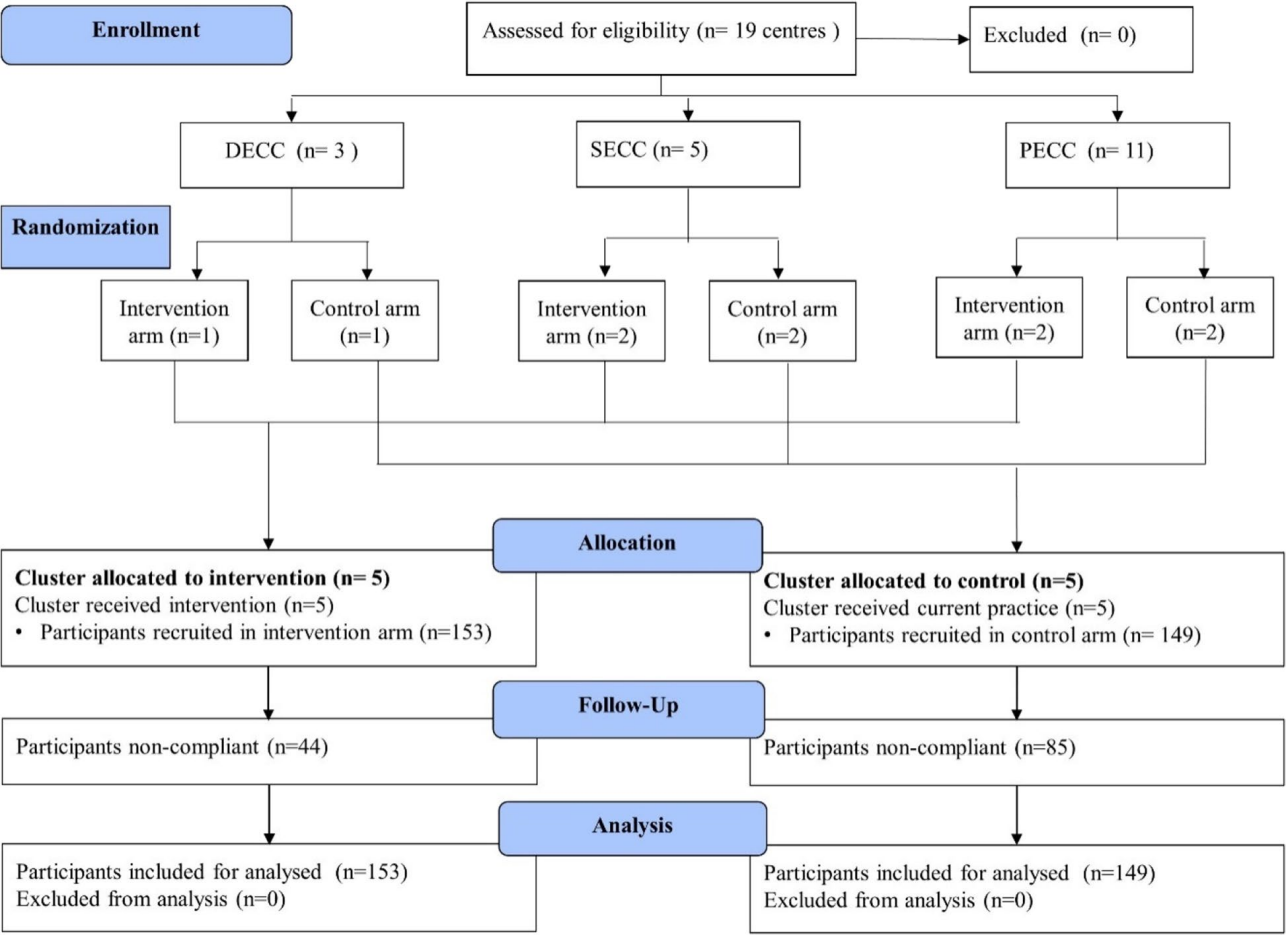
Recruitment of participants (Consort flow diagram)

The hospital records showed that the DECC referred 15-20 DR patients, SECC 7-10 DR patients and PECC 2-4 DR patients per month. With this flow rate of DR patients, we considered the sample size logistically feasible for the study duration.We projected that the ten recruiting centres would give us the appropriate sample size. We proposed to select an equal number of centres and populations from the hilly and ‘terai’ (marshy Himalayan foothills) regions. We used simple stratified random sampling to randomize the eye care centres. Out of three district eye care centres, we randomly selected two centres and randomized those into the control and intervention groups. Out of the five secondary eye hospitals, we randomly selected four and randomized them into the two groups. Out of 11 primary eye care centres, we randomly selected four and randomized equally into the control and intervention groups (Fig. 3). We used consecutive sampling at each centre to recruit the participants.

### Blinding

We did not mask the participants or the investigators.

### Data collection

At the referring centre, the trained counsellor recorded the socio-demographic and clinical information in the questionnaire. The counsellors also noted the referral information in the referral register. The counsellors coordinated with the referral manager at the LEIRC to document the compliance-related information. The investigators periodically visited the recruiting centres to review the recruitment process, data collection and management system.

### Statistical methods

The data entry was done by the researchers at Lumbini Eye Institute and Research Centre. We entered the data into a Microsoft Excel spreadsheet which was password protected and only accessible to the investigators. We analyzed the data using Stata, version 14. We compared the baseline information of the two groups by an appropriate parametric or non-parametric test based on the distribution. Referral compliance was estimated by proportion and 95% confidence interval (CI). We checked the difference in compliance by the test of proportion between the two groups. We estimated the mean difference in time to visit the higher centre between the two groups and tested the statistical significance by unpaired t-test. We performed sub-group analyses for the variables which are either significantly differing between the two groups after randomization and for clinically important variables. We considered a *p*-value of <0.05 as statistical significance for any difference between the two groups.

### Ethical consideration

We obtained the institutional ethics committee clearance of LEIRC (LEI/IRC/09/019/20). We obtained written informed consent from all the participants. For those who were uneducated or illiterate, we obtained written informed consent from first degree relatives/ guardians/ the closed ones. Additional permission was taken from all the participating institutes. The participants’ information is kept confidential. All the case report forms in hard copies have been kept in the research department of Lumbini Eye Institute and Research centre under lock and key for a minimum of five years. The entered data will be kept in the departmental password protected computers. If required, anonymized data will be shared with the partner organization and the funding agency.

## Results

We recruited a total number of 302 participants, 153 in the intervention arm and 149 in the control arm (Fig. 3). The mean age of the participants was 57.8 years (SD± 11.7 years). Both the arms were similar in terms of the socio-demographic variables including age, gender, religion, education and annual income (Table 1). The clinical characteristics of the two arms including smoking, diabetes, hypertension, cardiac disease duration of diabetes, presence of diabetic macular edema(DME) and visual acuity in the best eyes were also similar (Table 2).

**Table 1:** Sociodemographic status of the participants

**Characteristics**	**Intervention, n (%) (*N*=153)**	**Control, n (%) (*N*=149)**	***p*-value**
**Gender**			
Male	93 (60.8)	95 (63.8)	0.6
Female	60 (39.2)	54 (36.2)	
**Mean age (SD)**	58.0±11.7	57.5±11.8	0.7
**Religion**			
Hindu	131 (85.6)	128 (85.9)	0.6
Muslim	9 (5.9)	10 (6.7)	
Buddhist	12 (7.8)	8 (5.4)	
Christian	1 (0.7)	3 (2.0)	
**Occupation**			
Farmers	70 (45.7)	53 (35.6)	0.04
Business	41 (26.8)	36(24.1)	
Employed	24 (15.7)	25(16.8)	
Unemployed	18(11.8)	35(23.5)	
**Education**			
Illiterate	48(31.4)	36(24.2)	0.08
Primary	54(35.3)	57(38.2)	
Secondary	41(26.8)	34(22.8)	
Higher	10(6.5)	22(14.8)	
**Annual family income in lakh (In Nepali rupees)**			
<1.5	35 (22.9)	39 (26.2)	0.6
1.5-3.5	61 (39.9)	48 (32.2)	
3.5-5	25 (16.3)	28 (18.8)	
>5	32 (20.9)	34 (22.8)

**Table 2:** Clinical characteristics of the study participants

**Characteristics**	**Intervention, n (%) (*N*=153)**	**Control, n (%) (*N*=149)**	***p*-value**
**Smoking**			
Present	28(18.3)	26(17.5)	0.85
Absent	125(81.7)	123(82.5)	
**Hypertension**			
Present	64 (41.8)	78 (52.3)	0.07
Absent	89 (58.2)	71 (47.7)	
**Cardiac disease**			
Present	22 (14.4)	27 (18.1)	0.39
Absent	131 (85.6)	122 (81.9)	
**Duration of diabetes in years**			
<1	20 (13.1)	22 (14.8)	0.56
1-5	49 (32.0)	41 (27.5)	
5-10	30 (19.6)	38 (25.5)	
>10	54 (35.3)	48 (32.2)	
**Diabetic macular edema**			
Present	44 (28.8)	53 (35.6)	0.21
Absent	109 (71.2)	96 (64.4)	
**BCVA right eye**			
6/6- 6/18	106 (69.3)	93 (62.4)	0.33
6/18-6/60	27 (17.6)	26 (17.5)	
6/60- 3/60	11 (7.2)	13 (8.7)	
3/60- NPL	9 (5.9)	17 (11.4)	
**BCVA left eye**			
6/6- 6/18	105 (68.6)	96 (64.4)	0.67
6/18-6/60	36 (23.5)	35 (23.4)	
6/60- 3/60	6 (3.9)	9 (6.1)	
3/60- NPL	6 (3.9)	9 (6.1)	
**BCVA person wise best eye**			
6/6- 6/18	116 (75.8)	75 (69.2)	0.3
6/18-6/60	28 (18.3)	28 (18.8)	
6/60- 3/60	6 (3.9)	9 (6.0)	
3/60- NPL	3 (2.0)	9 (6.0)	

With implementation of SCREEN package, 71.2% (*n*=109) of the participants in the intervention group and 42.9% (*n*=64) the participants in the of the control group were compliant till three months of follow-up (*p*=0.001) (Table 3). The compliance was 28.3% (95%CI: 17.6, 39.0) higher in the intervention group (Power 99.8%). The compliance was 43% (*n*=66) with counselling alone in the intervention arm; however, the cumulative proportion increased to 66% (*n*=103) with first telephonic follow-up. The cumulative proportion did not improve much (*n*= 109, 71.2%) with the subsequent two follow-ups. We performed sub-group analyses in respect to occupation, low vision (visual acuity <6/18), and normal vision (>= 6/18) between the two arms. The compliance was higher (87.5%, 95% CI: 74.3,100.0) among those who are employed, in the intervention arm. The proportion of compliance was similar in all the subgroups in the respective arms for visual acuity and presence of systemic illness (Table 3).

**Table 3:** Subgroupanalysis of compliance to referral

**Group**	**Intervention**			**Control**		
	**Total Participant (n)**	**Referral compliant (n)**	**Proportion compliant (95% CI)**	**Total Participant (n)**	**Referral compliant (n)**	**Proportion compliant (95% CI)**
Overall	153	109	71.2 (64.1-78.4)	149	64	42.9 (35.0-50.9)
Occupation						
Farmers	70	46	65.7 (54.6-76.8)	53	22	41.5 (28.2-54.8)
Business	41	30	73.2 (60.0-87.3)	36	19	52.8 (36.5-69.1)
Employed	24	21	87.5 (74.3-100)	25	9	36.0 (17.2-54.8)
Unemployed	18	12	66.7 (44.9-88.4)	35	14	40.0 (23.8-56.2)
Visual acuity						
Normal	116	82	70.7 (62.4-79.0)	103	46	44.7 (35.1-54.3)
Low	37	27	73.0 (58.7-87.3)	46	18	39.1 (25.0-53.2)
Systemic disease						
Present	72	55	76.4 (66.6-86.2)	84	38	45.2 (34.6-55.9)
Absent	81	54	66.6 (56.4-76.9)	65	26	40.0 (28.1-51.9)

Among the compliant participants, the mean time taken by the participants to reach the referral centre was 14.7 days (SD± 9.4 days) and 18.2 days (SD± 9.1 days) in the intervention and the control arm respectively. Hence, on average, the compliant participants of the intervention arm reached 3.5 days early (95% CI: 0.7, 6.4) to the referred centre. The mean time taken by those who were compliant only with counselling was 8.2 days (SD± 3.0 days). The mean time taken in days after one telephonic follow-up was 23.1 days (SD± 4.3 days) and for two telephonic follow-ups was 34.3 days (SD± 3.4 days)

## Discussion

In this study, we aimed to improve the referral flow and compliance from the peripheral eye care centres to tertiary centre with the structured counselling and regular telephonic follow-up among the diabetic retinopathy patients in Nepal. We found that the compliance rate improved by 66% with this intervention compared to the current practice of unstructured follow-up. The intervention also shortened the average time gap between follow-up recommendation at the referral centre and reaching to the referred centre.

A systematic approach to improving the outcome of ophthalmic screening is a prerequisite for a health system. Screening must be coupled with targeted health education, timely referral for severe cases and provision of appropriate treatment [3].World Health Organization (WHO) has identified adherence to follow-up services as a critical component in the effective management of DR [11].It observed that improving the clarity of the referral process by explaining the treatment costs, the reason for referral and likely health benefits to the patient helped increase follow-up rates [11].Evidence from different parts of the world suggest that people often remain non-compliant with eye screening and treatment services because of several patient and provider-level factors [6]. The major patient-level challenges include ignorance of the condition, cost burden and sometimes perceived guilt of being unable to control blood sugar [6–9]. On the other hand, provider related reasons noted are the existence of poor counselling and advisory services for people with diabetes, long waiting times for screening or treatment, and complicated referral mechanisms or inaccessible locations where services are offered [6]. Evidence suggests that educating patients about DR is one of the key factors in improving the DR outcomes [14, 15]. Earlier evidence from Nepal showed that lack of awareness was the critical issue in service utilization for diabetic eye care [7, 12, 16]. Our findingsendorsed the same as compliance substantially improved with counselling alone. Further telephonic follow-up helped the participants to comply with the management plan. Telephonic follow-up found to be effective for diabetic retinopathy screening in resource-poor settings [17].We found that the improvement in compliance is uniformly distributed in all socio-economic and demographic strata. The only exception was the population who were employed had a substantially high compliance. This difference could be due to various reasons like provision of paid leaves and service-related insurance coverage [18].

The proportion of compliant participants with treatment alone was similar to the proportion of compliant in the control group except the fact that the time taken in the intervention arm was much less than the control arm. The additional gain in compliance in the intervention arm can be seen as the net improvement through counselling and telephonic follow-up. Evidence from similar settings suggests that the DR patients often delay in seeking referral care because of reasons like poor perception, lack of financial and social support [19]. Fortunately, the average delay in our study was much lesser than the reported delay of more than one year by another study conducted in 2014 [19]. The study also reported that nearly one-third participants required additional treatment because of the delay in referral follow-up. Hence, all efforts must be directed towards improving the follow-up compliance and time taken to follow-up. Apart from counselling and follow-up, other interventions like health system support, eye care advocacy, and creation of community support groups can be considered to improve compliance [20–22].

We have a few limitations of our study. First, the content of the counselling package was customized for this study but was not a previously validated package. However, we provided a checklist to ensure uniformity in data capture from the patients in the counselling session. Though not reported, we expected reporting bias as a few participants might have visited in any other health facilities. Furthermore, due to the limited scope of data collection, restricted solely to the health system, we were unable to account for potential confounding factors that could have influenced the study outcome.

### Generalisability

Our study results will be generalizable for development/ strengthening of referral system in similar low- income settings and geographic terrain, with some form of referral infrastructures in place.

## Conclusions

Counselling and follow-up of patients are the key factors to improve the utilization of the health services and the outcome of DR. Therefore, health system must be strengthened by optimizing the existing referral structure in Nepal to reduce the adverse outcome of diabetic retinopathy. Simultaneously, we must focus on generating evidence on other modalities of compliance improvement. We expect a similar benefit in referral compliance for the other ophthalmic and non-ophthalmic conditions through a structured counselling services in the primary health facilities in Nepal and other low-income countries.

### Supplementary Information

**Additional file 1:** **Supplementary Figure 1.** Flow of Patient at Referring Centre, Problem identified are written in blue. **Supplementary Figure 2.** Flow of Patient at Referral Hospital, Problems are written in Blue.

## Data Availability

Trial related anonymized data will be available from the corresponding author on reasonable request.

## Abbreviations

BCVA: Best Corrected Visual Acuity
B Scan: Brightness Scan.
COVID-19: Corona Virus Disease 2019
DECC: District Eye Care Center
DM: Diabetes Mellitus
DME: Diabetic Macular Oedema
DR: Diabetic Retinopathy
FFA: Fundus Fluorescence Angiography.
LASER: Light Amplification by the Stimulated Emission of Radiation.
LEIRC: Lumbini Eye Institute and Research Center
NPDR: Non Proliferative Diabetic Retinopathy
PDR: Proliferative Diabetic Retinopathy
PECC: Primary Eye Care Center
SECC: Secondary Eye Care Center
OPD: Out Patient Department.
OA: Ophthalmic Assistant
OCT: Optical Coherence Tomography
