# Supplementary material for: Structured Counselling and Regular Telephonic follow up to improve Referral flow and compliance in Nepal for Diabetic Retinopathy(SCREEN-D Study): a randomised controlled trial
Source: BMC Health Serv Res. 2024 Feb 10;24:188. doi: 10.1186/s12913-024-10647-3 (PMC10858536; doi:10.1186/s12913-024-10647-3)
Supplement: Supplementary file 1 — Additional file 1: Supplementary Figure 1. Flow of Patient at Referring Centre, Problem identified are written in blue. Supplementary Figure 2. Flow of Patient at Referral Hospital, Problems are written in Blue. [file 12913_2024_10647_MOESM1_ESM.docx]

**Supplementary Figure 1:** Flow of Patient at Referring Centre, Problem identified are written in blue.

Patient suggested for 6 monthly follow up and sent with glass, and advised to control diabetes mellitus.

**Counseling done by examiner**.

(No proper referral register maintained, no proper counseling done about the consequences of not going to higher center, unskilled counselor)

**DR Present**

**No signs of DR**

**Examination**

Visual acuity and posterior segment changes (hemorrhage, edema, new vessels)

**Optometrist/Ophthalmic Assistant**

(VA, BCVA, Slit Lamp examination of anterior segment, slit lamp or direct ophthalmoscope examination of posterior segment)

**Registration**

**Supplementary Figure 2:** Flow of Patient at Referral Hospital, Problems are written in Blue.

No counselor, No follow up, No tracking, No system of sending back to referring center.

**Examination done by Retina fellow/ retina surgeon.**

**Treatment planned and asked.**

**Severe, Very Severe NPDR and PDR are referred to room**

**Counseling done about the importance of follow up**

**Mild and Moderate NPDR**

**Grading of DR**

Mild, Moderate, Severe and Very Severe NPDR, PDR.

No referral registers

No fast track system

No consideration of referring centers OPD card

Patients undergo repeat examination by same kind of manpower which is not required.

**Room no 11: General ophthalmologist, Reevaluation of anterior and posterior segment with slit lamp and 90 D.**

**General OPD and repeat examination by OA (VA, BCVA, slit lamp examination of anterior segment, slit lamp examination or direct ophthalmoscope examination of posterior segment)**

**New cases arrive in room no 1 for new OPD registration.**
